# Induced abortion in villages of Ballabgarh HDSS: rates, trends, causes and determinants

**DOI:** 10.1186/s12978-015-0040-9

**Published:** 2015-05-29

**Authors:** Shashi Kant, Rahul Srivastava, Sanjay Kumar Rai, Puneet Misra, Lena Charlette, Chandrakant S. Pandav

**Affiliations:** Centre for Community Medicine, All India Institute of Medical Sciences, New Delhi, India

**Keywords:** Ballabgarh, Induced abortion, Abortion, Determinants

## Abstract

**Background:**

Induced abortion has been legal in India on a broad range of medical and social grounds since 1980s. Often, induced abortion is resorted to as a means for contraception, and has a potential to be misused for sex selective feticide. We assessed the rates, trends, causes and determinants of induced abortions from 2008–12 in a rural community of northern India.

**Methods:**

Present study is a secondary data analysis of pregnancy outcomes at Ballabgarh Health and Demographic Surveillance System from 2008–12. The data was retrieved from the Health and Management Information System maintained at Ballabgarh. Cause of abortion was self-reported by the women who underwent abortion.

**Results:**

Of the 11,102 pregnancies, 1,226 (11 %) culminated as abortions of which 425 (3.8 %) were induced abortions. Spontaneous abortion rate (7.2 %) was twice that of induced abortion rate (3.8 %). Both abortion rates had an increasing trend during the course of the study period. Self-reported reasons for opting for induced abortions were bleeding per vaginum (23 %), unwanted pregnancy (16 %), and unviable fetus diagnosed by ultrasonography (11 %). Eight percent of the induced abortions were due to the female sex of the fetus. About 11 % of the abortions were performed beyond 20 weeks of gestation which was the upper legal permissible gestational age for performing induced abortions in India. About 10 % of the abortions were performed by unqualified practitioners. Caste, wealth index, birth order and size of the village population were the factors that were significantly associated with induced abortion.

**Conclusions:**

Though the abortion rate was low, the proportionate contribution of induced abortion was more than what could be expected. Unsafe and sex selective abortion, though illegal, was prevalent. Upper caste and higher socio-economic status families were more likely to opt for induced abortion.

## Background

The global maternal mortality ratio in 2010 was 210 per 100,000 population [1]. Unsafe abortion accounted for 13 % of all direct causes of maternal death, next only to hemorrhage and infection [2]. Worldwide, in 2008, there were an estimated 43.8 million abortions: 22.2 million safe, and 21.6 million unsafe i.e. one in ten pregnancies ended as unsafe abortions. In 2008, there were 2 million more unsafe abortions in developing countries than in 2003. The global abortion rate was stable between 2003 and 2008, after a decline from 1995. But the proportion of unsafe abortions increased to 49 % in 2008, compared to 44 % in 1995. Unsafe abortions resulted in 47,000 maternal deaths, and 8.5 million women suffered from the complications due to unsafe abortions in 2005 [3–5].

The World Health Organization defines abortion as termination of pregnancy before the foetus attains viability. Induced abortions are those terminated by deliberate action undertaken with the intention of terminating pregnancy. Spontaneous abortions are all other abortions, even if an external cause such as trauma or communicable disease is involved. Unsafe abortion is defined as a procedure for terminating an unintended pregnancy carried out either by persons lacking the necessary skills or in an environment that does not conform to minimal medical standards, or both [6].

The reasons for abortions are varied and include socioeconomic concerns, family building preferences, religious factors, service availability, legal status of abortion, unwanted pregnancies, and risk to maternal or fetal health. However, the major underlying causes are unawareness or lack of access to contraceptives [7, 8]. The determinants of induced abortion include residence in urban areas, higher education of women, high standard of living, women who begin cohabiting at higher ages, and who had fewer living children. Sex of previous child was not significantly associated with induced abortion [9]. Although abortion done in appropriate health facility are relatively free of complications, unsafe abortions contribute significantly to mortality and morbidity, affecting subsequent fertility and causing psychological distress [6, 7].

In India, abortion was legalized since 1971. Pregnancies up to 20 weeks could be terminated if it entailed a risk to the life of the mother, in case of pregnancy due to rape or contraceptive failure, and in case of physical or mental abnormality in child [10].

Several studies have estimated the induced abortion rate to be between one to seven per 100 pregnancies [11–16]. Little was known about the causes and factors determining induced abortions in the study area. Present study was conducted to ascertain the rates, trends, causes and determinants of induced abortions in a rural community of Haryana state in northern India.

## Materials and methods

We conducted retrospective analysis of records maintained in Health and Management Information System (HMIS) of Ballabgarh Health and Demographic Surveillance System (HDSS). The records pertained to the population residing in 28 villages of Ballabgarh also known as Ballabgarh HDSS, located in district Faridabad of state Haryana in northern part of India. At Ballabgarh HDSS, the population was under surveillance since 1967 [17]. The project was a collaboration between the All India Institute of Medical Sciences (AIIMS), New Delhi, and the State Government of Haryana. Total population under Surveillance was 92,070 (31^st^ December 2012) and was served by two Primary Health Centres (PHCs) and 12 sub-centres. A 13 digit unique identification number existed for every person residing in the study area for more than six months.

The Multipurpose Health Worker (Female) posted at the sub-centre, made monthly domiciliary visits to each house following a pre-fixed schedule. During these visits, apart from delivering health services to the residents, workers collected various information pertaining to health and demography, which included information on eligible couple, pregnancies and their outcome, including abortions. Effort was made to register the pregnancies as soon as possible. Internal review of records had revealed that > 95 % of pregnancies were registered before 12 weeks of gestation. The information collected was recorded in a database maintained at Ballabgarh. For quality assurance, 10 % and 5 % of the recorded information were routinely and randomly re-checked by Health Supervisors and Medical Officers respectively. Information regarding abortion status was being routinely collected since 2008. We analyzed the data from 1^st^ January 2008 to 31^st^ December 2012. The outcome of all registered pregnancies was studied including self reported induced abortions, irrespective of gestational age or the place at which induced abortion was performed.

Present study was a secondary data analysis of a surveillance data and hence prior permission was taken from the in-charge of the HDSS. Study team was blinded for the personal identifiers of the women who underwent abortion.

Statistical Analysis: Data was analyzed using STATA (Release 9, StataCorpLP, College Station, TX, USA). The Induced Abortion Ratio (Induced abortions per 100 live births) and Induced abortion Rate (Induced abortions per 100 pregnancies) were calculated. A p-value of <0.05 was considered statistically significant. The 95 % confidence intervals (CI) for proportions, induced abortion ratio, and odds ratios were calculated. Trend analysis tool of Microsoft office excel 2007 was used for adding the trend line (linear equation; y = mx + c) and calculating R^2^. The association between caste, wealth index, village population, sex ratio (nos. of female per 1000 males), availability of Primary Health Centre/Sub-centre in the village, maternal age at delivery, paternal age, parental education, and birth order with induced abortion were examined (live births Vs induced abortion). All independent variables with *p* < 0.25 were selected for inclusion in logistic regression. Logistic regression was performed using these selected independent variables and induced abortion as an outcome. Caste was categorized into three categories – upper caste (general caste), middle caste (backward caste), and lower caste (schedule caste and schedule tribes). Wealth Index used in the present study was of the same methodology as used in National Family Health Survey (NFHS) III [18]. It is a pure economic variable that measures the relative economic standing. It uses information on household assets and utility services to construct a score with relative weights derived through Principal Component Analysis. During analysis the wealth index was divided into three categories – Higher, middle and lower socio-economic status.

## Results

**Fig. 1:**
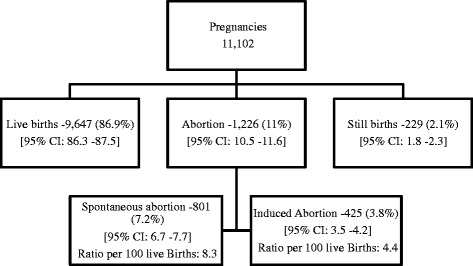
Pregnancies with outcome in Ballabgarh HDSS (2008–12)

During 2008–12, a total of 11,102 pregnancies were registered in the study area, of which 9,647 (86.9 %) were live births and 1,226 [11 % (95 % CI: 10.5 % – 11.6 %)] culminated in abortions. The incidence of spontaneous abortion was 7.2 % (95 % CI: 6.7 %–7.7 %). The incidence of induced abortion was 3.8 % (95 % CI: 3.5 %–4.2 %), and the Induced abortion ratio was 4.4(95 % CI: 3.8–4.6 %) per 100 live births (Fig. 1).

**Fig. 2:**
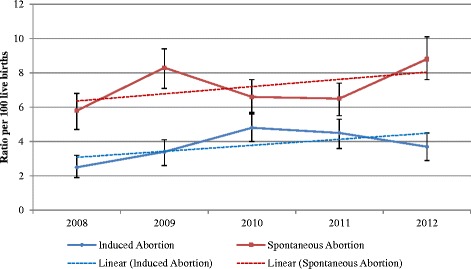
Trend in abortion rates (Induced and Spontaneous), 2008–12

Higher ratio of induced abortions were observed among upper caste (5.0), higher wealth index (5.4), paternal age >30 years, (5.6), maternal age >30 years (7.0), and birth order of four or more (8.2) (Table 1). About 80 % of induced abortions were carried out by private doctors at private health facility, and 10 % of abortions were conducted at home by nurses or *Dais* (trained birth attendants). About 11 % of induced abortions were performed beyond 20 weeks of gestation. Among the pregnancies terminated beyond 20 weeks, 71 % (17) were terminated at home and the rest at a facility. About 54 % of the induced abortions were performed among women who had no living male child, while 34 % had no living female child. Induced abortion ratio was almost same in different strata of maternal and paternal education (Table 1). An increasing trend (trend line) was observed for induced and spontaneous abortion ratios over the years (Fig. 2). The Chi - square for trend was not statistically significant (*p* = 0.47).

**Table 1:** Characteristics of live births and induced abortions

Variable	Sub group	Percentage of live births	Percentage of induced abortions	Induced abortion ratio (per 100 live births)	95 % CI
Caste	Upper	30.2	34.3	5.0	4.2 – 5.8
	Middle	43.5	46.9	4.7	4.1 – 5.4
	Low	26.3	18.8	3.1	2.5 – 3.9
Wealth index	High	36.9	44.8	5.4	4.7 – 6.3
	Middle	32.3	30.3	4.2	3.5 – 5.0
	Low	30.9	24.9	3.6	3.0 – 4.4
Paternal age	<20	2.3	2.6	5.0	2.7 – 8.9
	20–24	23.9	15.5	2.8	2.3 – 3.7
	25–29	40.5	40.3	4.4	3.8 – 5.1
	≥30	33.2	41.5	5.6	4.8 – 6.4
Maternal age	<20	8.6	6.6	3.4	2.3 – 4.9
	20–24	55.7	43.1	3.4	3.0 – 3.9
	25–29	24.9	33.2	5.9	5.0 – 6.9
	≥30	10.8	17.2	7.0	5.6 – 8.7
Paternal education	Low	64.5	64.5	4.4	4.0 – 5.0
	High	35.5	35.5	4.4	3.8 – 5.2
Maternal education	Low	80.8	83	4.5	4.1 – 5.0
	High	19.2	17	3.9	3.1 – 4.9
Birth order	1	38.3	18.4	2.1	1.7 – 2.7
	2	32.6	29.4	4.0	3.4 – 4.8
	3	17.3	30.4	7.8	6.6 – 9.2
	≥4	11.9	21.9	8.2	6.7 – 10.0
Place of delivery	Govt. hospital	36.6	7.3	0.8	0.6 – 1.2
	Private	35.8	79.1	9.7	8.8 – 10.8
	Home	27.0	13.2	2.1	1.7 – 2.8
	Others	0.6	0.5	3.2	0.2 – 11.7
Personnel conducting the delivery	ANM/Nurse	2.2	8.0	16.3	11.8 – 21.9
	Trained birth attendant	26.8	1.2	0.2	0.1 – 0.5
	Untrained birth attendant	0.2	-	-	-
	Govt. doctor	34.9	7.3	0.9	0.6 – 1.3
	Private doctor	35.5	78.8	9.8	8.8 – 10.8
	Others	0.5	4.7	43.5	30.2– 57.8
Gestational age	≤12	-	54.8	-	-
	13-20	-	34.1	-	-
	>20	-	11.1	-	-
No of male siblings	0	-	54.4	-	-
	1	-	35.1	-	-
	2	-	8.7	-	-
	3-5	-	1.8	-	-
No of female siblings	0	-	33.6	-	-
	1	-	36.9	-	-
	2	-	20.0	-	-
	3	-	7.1	-	-
	4-6	-	2.3	-	-

According to self-reported cause for abortion, “Bleeding per vaginum” (23 %) was the most common, followed by “unwanted pregnancy” (16 %), and “fetal demise” (11 %), as ascertained by absent or low heart rate in Ultrasonography. Sex selection as a cause of abortion was reported by 8 % and was the fourth leading cause for inducing an abortion (Table 2). There were no multiple options recorded.

**Table 2:** Distribution of self reported cause for undergoing induced abortions (n = 425)

Cause	Frequency	Percentage
Bleeding PV	98	23.1
Unwanted pregnancy	68	16.0
Dead foetus	46	10.8
Sex selection	35	8.2
Congenital malformation	26	6.1
Growth retardation	13	3.1
Tubal pregnancy, Less heart rate	2	0.4
No response	137	32.2
Total	425	100

In bi-variate analysis, caste, wealth index, size of village population, sex ratio in the village, paternal age, maternal age and birth order were found to be significantly associated with induced abortion. After adjusting for other factors, caste, wealth index, size of village population and birth order were found to be the factors determining induced abortions. The odds of having an induced abortion was lesser [(0.63, 95 % CI: 0.47 – 0.86)] among lower caste when compared to upper caste. The odds of having an induced abortion also declined with the declining socioeconomic status as reflected by the wealth index. The odds ratio was 0.73 (95 % CI: 0.57–0.93) in middle and 0.62 (95 % CI: 0.47 to 0.81) in low socio economic groups as compared to the high socio economic group. The risk of induced abortion was 1.9 times higher in villages having moderate population size (3,000 – 6,000) as compared to villages having population <3000. With birth order, the induced abortion risk rose and was highest in birth order more than four (Table 3).

**Table 3:** Determinants of induced abortion

Variable	Subgroups	OR	95 % CI	Adjusted OR	95 % CI
Caste	Upper	Ref*	Ref	Ref*	Ref
	Middle	0.95	0.76 – 1.18	1.00	0.79 – 1.28
	Low	0.63*	0.48 – 0.83	0.63*	0.47 – 0.86
Wealth Index	High	Ref*	Ref	Ref*	Ref
	Middle	0.78*	0.61 – 0.98	0.73*	0.57 – 0.93
	Low	0.67*	0.52 – 0.86	0.62*	0.47 – 0.81
Village population	<3000	Ref**	Ref	Ref**	Ref
	3000–6000	1.90**	1.53 – 2.38	2.27**	1.72 – 3.00
	>6000	0.62*	0.47 – 0.81	0.7*	0.51 – 0.95
Sex Ratio	<800	1.30*	0.87 – 1.88	0.68	0.42 – 1.08
	800–1000	0.88	0.61 – 1.26	0.82	0.55 – 1.25
	>1000	Ref*	Ref	Ref	Ref
Availability of PHC/SC	No	Ref	Ref		
	Yes	1.11	0.91 – 1.37		
Maternal age		1.02*	1.01 – 1.03	0.93	0.79 – 1.10
Paternal age		1.06**	1.04 – 1.08	0.97	0.83 – 1.13
Parental education	Both low	Ref	Ref	-	-
	Paternal low	1.04	0.65 – 1.68	-	-
	Maternal low	1.12	0.88 – 1.42	-	-
	Both high	0.84	0.62 – 1.14	-	-
Birth order	1	Ref**	Ref	Ref**	Ref
	2	1.89**	1.41 – 2.51	2.12**	1.56 – 2.89
	3	3.65**	2.74 – 4.87	4.22**	3.02 – 5.89
	≥4	3.84**	2.83 – 5.23	5.38**	3.6 – 8.02

*p value between 0.001 & 0.05.**p value <0.001

## Discussion

The overall abortion rate (11 %) in the present study was lower than the figures recorded in 1984 [14] for the same study area (15.5 %) or for the state of Haryana (15 %) recorded for the 5 year period of 1993–98 [16]. The proportion of pregnant women who reported unwanted pregnancy as a reason for induced abortion was low (16 %). It is therefore possible that the observed decline in overall abortion rate is a reflection of greater access to contraceptive services.

We found that the spontaneous abortion rate was almost twice that of the induced abortion rate. Also for entire Haryana state (study site) the spontaneous abortion rate was thrice of induced abortion rate (10.7 % Vs 3 % respectively) [19]. This is an intriguing finding. Globally, the spontaneous abortion rates are lower as compared to induced abortion rate [3]. Hence, what could possibly explain such reversal of proportion between spontaneous and induced abortion rates?

It is possible that some of the registered pregnancies later on opted for sex selective induced abortion. In almost one-third of induced abortion instances, no reason was proffered by the women. Could it be that all or majority of these cases were sex selective induced abortion? Strong preference for male child and therefore resorting to sex selective abortion is a reality in our study area. During the study period, the number of females per 1000 males at birth in study area ranged between 870 and 787. In this study 54 % of induced abortions were performed among women who had no living male child. In the district where the study was conducted, the number of females per 1000 males at birth during the period 1994–2000 was 873 which further worsened to 860 during the period 2004–10 [20]. We therefore believe that sex selective induced abortion was more widespread than the observed figure of 8.2 %. Women do not report induced abortion primarily because of the stigma associated with induced abortion [21]. Some women might misconstrue induced abortion as a criminal offence and thus intentionally misreport it as spontaneous abortion [22]. Thus, due to stigma and/or perceived illegality, some of these sex selective induced abortions could have been deliberately misreported as spontaneous abortion. Such a situation could reverse the expected proportion of spontaneous abortion.

Upper caste which denoted high relative position in social hierarchy and a proxy for greater access to health care; and higher wealth index denoting financial wherewithal were risk factors for induced abortion. In our study, about 80 % of the induced abortions were carried out at a private health facility denoting ability to pay for such services. Thus, women with requisite resources (social, financial, etc) were able to avail induced abortion.

Our findings were supported by other studies [9, 13]. In general, majority of the abortions in India took place at private facility, which had financial implications [11]. The out of pocket expenditure incurred ranged from INR 800 to INR 4,000, much higher in private facilities than in public facilities [14, 22]. This might partly explain the higher rate of induced abortion among higher socio-economic classes and in upper caste.

The strengths of the study are that all eligible couples in the study area were listed and visited every month to collect data prospectively. Nearly 85 % of all pregnancies in the study area had been registered before 12 weeks of gestation. The pregnancies were prospectively followed up and the details regarding pregnancy outcome, including abortions when reported, were obtained within a month of the incident. This may have helped in reduced recall bias.

We assume that social desirability bias was minimized since the multipurpose worker (female) who collected data enjoyed the confidence of the participants due to their long standing service provision. Our assumption is supported by the observation that participants were willing to disclose sex selective abortion, which is otherwise illegal and prohibited by the law. No self-reported cause of induced abortion was provided by one third of the women. In the worst case scenario, assuming all of them had resorted to induced abortion due to the sex of the fetus, the situation becomes extremely alarming. Although a secondary data analysis, the process of data collection was uniform in a standard format across subcentres and the same was verified by the health assistants and medical officers of the PHC.

Few pregnancies might have been terminated without being disclosed to the health workers. This would be particularly true in situation where prenatal sex determination was contemplated. This would have resulted in an underestimate of the overall abortion rate. A second limitation was that some degree of misclassification between spontaneous and induced abortions was possible. This could be due to deliberate misreporting by the study subjects. Though this would not affect the overall abortion rate, but the rate for different type of abortion would be affected. We did not get any self- reported reason for almost one-third of induced abortion cases, and thus were forced to speculate about its putative cause.

## Conclusions

The incidence of spontaneous and induced abortion rate was 7.2 % and 3.8 % respectively. Abortion rate showed an increasing trend over the years. Upper caste and richer families were more likely to opt for induced abortions in comparison to lower caste and poorer families. No self-reported cause could be elicited in one third of the induced abortion cases. Sex selective abortion though illegal, was still practiced.

### Summary of conclusions, strength and limitations

The spontaneous and induced abortion rates were 7.2 and 3.8 per 100 pregnancies. The abortion rates had been increasing over the years. Self-reported cause in one third of the abortion cases could not be elicited. Unwanted pregnancies and sex of the fetus were among the four major causes of induced abortions. Upper caste, higher socioeconomic status, moderate village population size (3,000–6,000) and higher birth order (>3) were found to be significantly associated with higher odds of induced abortions.

The strength of the study was that all registered pregnancies in the study area were followed up prospectively for their outcome by trained workers.

The limitations were: A few early pregnancies might have been terminated without being registered, and some degree of misclassification between spontaneous and induced abortions was possible. Stigma associated with induced abortion could be a contributory factor for both limitations.
